# Telomere length as a predictor of emotional processing in the brain

**DOI:** 10.1002/hbm.24487

**Published:** 2018-12-04

**Authors:** Timothy R. Powell, Simone De Jong, Gerome Breen, Cathryn M. Lewis, Danai Dima

**Affiliations:** ^1^ Social, Genetic and Developmental Psychiatry Institute of Psychiatry, Psychology and Neuroscience, King's College London London United Kingdom; ^2^ National Institute for Health Research Biomedical Research Centre for Mental Health Institute of Psychiatry, Psychology and Neuroscience at the Maudsley Hospital and King's College London London United Kingdom; ^3^ Department of Medical and Molecular Genetics Guy's Hospital, King's College London London United Kingdom; ^4^ Department of Psychology, School of Arts and Social Sciences, City University of London London United Kingdom; ^5^ Department of Neuroimaging Institute of Psychiatry, Psychology and Neuroscience, King's College London London United Kingdom

**Keywords:** bipolar disorder, emotional faces, fMRI, polygenic risk score, telomere length

## Abstract

Shorter telomere length (TL) has been associated with the development of mood disorders as well as abnormalities in brain morphology. However, so far, no studies have considered the role TL may have on brain function during tasks relevant to mood disorders. In this study, we examine the relationship between TL and functional brain activation and connectivity, while participants (n = 112) perform a functional magnetic resonance imaging (fMRI) facial affect recognition task. Additionally, because variation in TL has a substantial genetic component we calculated polygenic risk scores for TL to test if they predict face‐related functional brain activation. First, our results showed that TL was positively associated with increased activation in the amygdala and cuneus, as well as increased connectivity from posterior regions of the face network to the ventral prefrontal cortex. Second, polygenic risk scores for TL show a positive association with medial prefrontal cortex activation. The data support the view that TL and genetic loading for shorter telomeres, influence the function of brain regions known to be involved in emotional processing.

## 1 INTRODUCTION

Biological aging, as opposed to chronological aging, is the concept that two individuals born on the same date, may be aging at different rates at any one particular time, at the cellular level (Jin, 2010). Telomeres are repeat DNA sequences at the end of chromosomes that shorten upon each cell division, and relative telomere length represents a commonly used measure of cellular age (Stewart, Chaiken, Wang, & Price, 2012). Characteristically, older individuals who have experienced a greater number of cell divisions have shorter telomere lengths (TLs), relative to younger individuals (Saretzki & von Zglinicki, 2002). However, shorter TL relative to one's chronological age, is associated with increased risk for age‐related diseases, and both environmental and genetic factors have been found to moderate the rate of TL shortening (Shammas, 2011).

In the realm of psychiatry, TL is becoming of increasing interest after the realization that TL shortening appears to be accelerated in those who have experienced maltreatment and those with psychiatric disorders (Drury et al., 2012). Faster shortening of telomeres has been found after life stress (Epel et al., 2004), exposure to violence during childhood (Shalev et al., 2013), experiencing highly disadvantaged environments (Mitchell et al., 2014) and early life adversity (Ridout et al., 2018). Short TL in psychiatry has been linked primarily to patients suffering from major depressive disorder (Hartmann, Boehner, Groenen, & Kalb, 2010; Lung, Chen, & Shu, 2007; Monroy‐Jaramillo, Dyukova, & Walss‐Bass, 2017; Ridout, Ridout, Price, Sen, & Tyrka, 2016; Schutte & Malouff, 2015), post‐traumatic disorder (Lindqvist et al., 2015; Shalev et al., 2013), anxiety disorders (Monroy‐Jaramillo et al., 2017; Shalev et al., 2013) and bipolar disorder (Barbé‐Tuana et al., 2016; Lima et al., 2015). However, not all patients have shortened TL, and evidence suggests that TL may actually be marking the effects of specific environmental risk factors for psychiatric disorders, or the severity of specific cognitive biases or symptoms, rather than representing a definitive case/control predictor (Elvsåshagen et al., 2011; Powell, Dima, Frangou, & Breen, 2018; Vincent et al., 2017). One of the key cognitive biases associated with mood disorders is in the way patients process emotion, while demonstrating altered patterns of brain activation compared with healthy controls (Mourão‐Miranda et al., 2012; Stuhrmann, Suslow, & Dannlowski, 2011). Interestingly, functional magnetic resonance imaging (fMRI) studies have revealed regional differences in brain activation during reading of facial emotions between young and old participants, (Ebner, Johnson, & Fischer, 2012), suggesting the way we process emotion may be subject to the effects of age. Premature biological aging may, therefore, affect or predict, the way an individual processes emotion, and consequently their susceptibility to developing a mood disorder.

Although the reasons behind functional changes to the normal brain as we age are likely complex, there is relevant research linking TL with the health and function of neurons. Literature to‐date suggests that factors associated with shorter TL such as heightened cortisol, decreased mitochondrial number, immuno‐inflammatory activation, and oxidative stress can dynamically influence neuron survival, synapse formation, the generation of action potentials, and region‐specific differences in brain volume (Epel & Prather, 2018; King et al., 2014; Mamdani et al., 2015; Mottahedin et al., 2017; Nilsonne, Tamm, Månsson, Åkerstedt, & Lekander, 2015; Powell et al., 2018; Wang & Michaelis, 2010; Warren et al., 2018). Consequently, environmental stressors may cumulatively impact upon TL, the health of neurons, and the functional reorganization of the brain as we age.

As well as telomeres representing a potential *state* biomarker capturing environmentally mediated stress on the brain at a given period of time, studies suggest that genetic regulators of TL may represent *trait* biomarkers. Our recent work revealed that the strongest genetic risk variant for shorter TL directly increases risk for childhood‐onset major depression in a UK sample, suggesting that for some mood disorder subtypes TL may play a causal role (Vincent et al., 2017). Although the reason behind this association needs to be verified, both in vitro and nonhuman animal studies have shown that shorter TL during development inhibits the proliferation and differentiation of neural stem cells, and consequently could evoke enduring differences to neural morphology, organization and connectivity, relevant to mood disorders (Ferron et al., 2009; Liu, Nemes, & Zhou, 2018; Zhou et al., 2016). Indeed, animal studies have shown that a genetic deficit in the telomere lengthening enzyme telomerase has drastic effects on the brain and evokes depression‐like behaviors (Zhou, Ning, Lee, Hambly, & McLachlan, 2016).

Consequently, TL itself may possess state biomarker properties, encapsulating the influences of the environment as we age, on brain functionality, whereas genetic risk for telomere length alone, may represent a trait biomarker whereby it captures the enduring neurodevelopmental effects of genetic risk on brain organization and function. In this study, we attempt to better understand the relationship between TL and functional brain activation and connectivity, by studying participants (controls, relatives of BD subjects, and BD subjects) performing an fMRI facial affect recognition task. In addition, we generated polygenic risk scores for TL (PRS‐TL), which encapsulates single nucleotide polymorphisms (SNPs) that predict TL, into an individualized score. We used PRS‐TL to better understand whether genetic risk for shorter telomeres could represent a trait biomarker for altered face‐related activation and connectivity.

## 2 MATERIALS AND METHODS

### 2.1 Participants

Buccal DNA was available from 217 individuals of White British ancestry who had participated in the Vulnerability to Bipolar Disorders Study (VIBES), described previously (Frangou, 2009; Powell et al., 2018). The sample comprised 63 patients with BD, 74 first‐degree relatives (siblings = 35; offspring = 39) and 80 unrelated healthy volunteers (Supporting Information Table S1). The telomere length was significantly negatively associated with age in the entire sample (beta = −0.18; *t* = −2.72; *p* = .007) but not with sex (beta = 0.07; *t* = 1.09; *p* = .29). From those, 112 underwent a neuroimaging session; the final study sample comprised euthymic patients with BD (n = 41), their healthy first‐degree relatives (n = 25), and demographically matched unrelated healthy individuals (n = 46) (Table 1). All participants were of White British ancestry. They were assessed using the structured clinical interview for the Diagnostic and Statistical Manual of Mental Disorders, 4th edition, revised (DSM‐IV) for Axis I diagnoses (First, Spitzer, Gibbon, & Williams, 2002a, 2002b). Patients that fulfilled the criteria for BD, type I according to the DSM‐IV (American Psychiatric Association, 1994) were included. The relatives were carefully selected from the VIBES sample based on the absence of any lifetime history of psychopathology. The sample included 17 BD patients‐sibling pairs from 17 different families. Unrelated healthy individuals were selected based on the absence of family history and personal lifetime history of psychiatric disorders. In all participants, current IQ was assessed using the Wechsler Adult Intelligence Scale 3rd Edition (Wechsler, 1997) and psychopathology was rated using the Hamilton Depression Rating Scale (Hamilton, 1960) (HDRS), Young Mania Rating Scale (Young, Biggs, Ziegler, & Meyer, 1978) (YMRS) and Brief Psychiatric Rating Scale (Lukoff, Nuechterlein, & Ventura, 1986) (BPRS). Psychopathology was assessed weekly in patients over a period of 1 month prior to testing and at each assessment they scored below 7 in the HDRS and YMRS. Patients were also required to have remained on the same type and dose of medication for a minimum of 6 months. The BPRS, HDRS, and YMRS scores were highly correlated (all *r* > 0.73, *p* < .0001). To avoid collinearity, we used the total BPRS score as a covariate in subsequent neuroimaging analyses because, unlike the two other scales, it is applicable to nonclinical populations.

**Table 1:** Demographic, clinical, and behavioral data

	BD patients (n = 41)	HI (n = 46)	Healthy relatives (n = 25)
Age	44.3 (11.9)	40.3 (13.2)	39.7 (13.7)
Sex (male/female)	20/21	25/21	13/12
IQ	117.9 (17.9)	112.6 (14.5)	115.8 (18.5)
HDRS total score^a^	4.8 (5.3)	0.1 (0.5)	0.14 (0.4)
YMRS total score^a^	1.4 (3.0)	0.2 (0.6)	0.0 (0.0)
BPRS total score^a^	27.5 (4.0)	24.3 (0.7)	24.1 (0.4)
Telomere length	1.63 (0.58)	1.69 (0.53)	1.49 (0.58)
Age of onset (years)	24.7 (8.0)	–	–
Duration of illness (years)	20.2 (10.5)	–	–
Depressive episodes (n)	5.7 (7.5)	–	–
Manic episodes (n)	5.6 (7.7)	–	–
Lithium (n)	12	–	–
Any medication (n)	38	–	–
Any antidepressant (n)	9	–	–
Any antipsychotic (n)	8	–	–
Any anticonvulsant	13	–	–
Correctly identified emotional faces, %	90.3 (4.1)	93.1 (4.8)	90.1 (5.2)
Response time to emotional faces, s^b^	1.4 (0.20)	1.10 (0.24)	1.09 (0.14)

Unless otherwise indicated, data are expressed as mean (standard deviation). Bipolar disorder (BD); Healthy individuals (HI); number (n); seconds (s); Hamilton Depression Rating Scale (HDRS); Young Mania Rating Scale (YMRS); Brief Psychiatric Rating Scale (BPRS); Global Assessment of Functioning (GAF).

a Scores for BD patients are significantly greater than those for HI and unaffected first‐degree relatives (*p* < .019).

b BD patients had longer mean response times compared with HI and to unaffected first‐degree relatives (*p* < .009).

The study was approved by the Joint South London and Maudsley and Institute of Psychiatry research ethics committee. All participants provided written informed consent before study participation.

### 2.2 Telomere length assessment

Buccal DNA was extracted using a standardized procedure described previously (Freeman et al., 2003). DNA samples had good purity ratios (260/280 ratios of between 1.7 and 1.9), as measured using the Nanodrop, ND1000 (Thermoscientific, Wilmington, DE). Telomere length was quantified using the output from two separate quantitative real‐time polymerase chain reactions (qPCRs). The first qPCR assays the telomere repeat region (TTAGGG), and the second qPCR assays a single copy gene (albumin). The ratio between the telomere repeat region and the single copy gene was calculated to determine relative TL. Five calibrator DNA samples were included in every plate to account for inter‐plate variability, see Powell et al. (2018) for further details. Both reactions were performed on the ABI Prism 7900HT Sequence Detection System, with the output generated using SDS Software version 2.2.

### 2.3 Polygenic risk score telomere length (PRS‐TL) construction

DNA extracted from buccal swabs was genotyped on the Psych Chip (Illumina Infinium PsychArray‐24). Data quality was controlled in PLINK v1.07 (Purcell et al., 2007) using the same parameters as described in Coleman et al. (2016) and Dima, de Jong, Breen, and Frangou (2016). To generate polygenic risk scores for telomere length (PRS‐TL), we obtained the genome‐wide association study (GWAS) summary statistics from the largest TL GWAS to‐date (Codd et al., 2013). SNP positions were lifted over from hg18 to hg19 build using UCSC LiftOver tool (Kuhn, Haussler, & Kent, 2013).

To obtain the optimal *p*‐value threshold with which to generate our PRS‐TLs, we utilized corresponding genetic and TL data (adjusted for age and sex) from all unrelated individuals in the full VIBES sample (n = 136). Polygenic risk scores were generated within PRSice (Euesden, Lewis, & O'Reilly, 2015), whereby, we identified the optimal *p*‐value threshold in our base dataset (TL GWAS summary data), which explained the most variance in adjusted TL in VIBES, our target dataset. Subsequently, we output individualized PRS‐TL for the 112 individuals with fMRI data at this *p*‐value threshold.

### 2.4 Facial affect recognition paradigm

Three negative facial emotions (fear, anger, and sadness) were examined in three separate event‐related experiments presented in a random order during a single acquisition session. Each experiment lasted for 5 min. In each experiment, 10 different facial identities (six female, four male; http://www.paulekman.com) depicting 150% intensity of an affective or a neutral facial expression were used. Faces were presented in alternation with a fixation cross in a pseudorandom order. The fixation cross, neutral faces and affective faces were each displayed for 2 s and repeated 20 times (each facial identity was shown twice as a neutral expression and twice with an effective expression), giving a total of 60 images in each experiment. The inter‐stimulus interval followed a Poisson distribution and was varied between 3 and 9 s (mean interval, 5 s). Participants were instructed to press the right or left button with their dominant hand on an MRI‐compatible response box to indicate whether the face was affective or neutral in each trial. Participants were familiarized with the task off‐line 1 hr before the scan. Response time and accuracy data were collected.

### 2.5 Image acquisition

Anatomical and functional imaging data were acquired during the same session using a General Electric Sigma 1.5 Tesla. A high‐resolution T1‐weighted structural image was acquired for each participant in the same session in the axial plane for co‐registration (inversion recovery prepared, spoiled gradient‐echo sequence; repetition time = 18 ms, echo time = 5.1 ms, flip angle = 20°, slice thickness = 1.5 mm, matrix size = 256 × 192, field of view = 240 × 180 mm, voxel dimensions = 0.9375 × 0.9375 × 1.5 mm).

For the facial affect recognition paradigm, 450 T2‐weighted MR images reporting blood‐oxygen‐level dependent (BOLD) contrast were acquired (repetition time = 2,000 ms, echo time = 40 ms, flip angle = 70°, slice thickness = 7 mm, matrix size = 64 × 64, voxel dimensions = 3.75 × 3.75 × 7.7 mm).

### 2.6 Functional neuroimaging data analysis

Data were analyzed in SPM8 (http://www.fil.ion.ucl.ac.uk/spm/software/spm8/). Data from each paradigm were analyzed separately. FMRI images were realigned, normalized, and smoothed using an 8‐mm full‐width‐half maximum Gaussian kernel. Each participant's fMRI data from the three event‐related experiments (fear, anger, or sadness) were concatenated and vectors of onset representing correct responses were convolved with a canonical hemodynamic response function. Six movement parameters were also entered as nuisance covariates. The means of the three sessions as well as the transition at the end of each session were also modeled and images for the affect > neutral faces contrast were produced for each participant. Contrast images from each participant were entered into second‐level analyses using a one‐sample *t*‐test to identify clusters of increased task‐related activation with a family wise error (FWE) peak‐level whole‐brain corrected *p* < .05 and minimum cluster size (*k*) > 20. The BPRS total score and familial relatedness were added as covariates. The effect of group (patients, relatives, and controls) was tested using a one‐way analysis of variance with the BPRS total score as covariate. Suprathreshold clusters were identified using a FWE whole‐brain corrected peak‐level of *p* < .05, *k* > 20.

### 2.7 Effect of TL and PRS‐TL on face‐related activation

Contrast images from each participant were entered into second‐level analysis of regression in SPM8 to identify clusters of correlation with TL and PRS‐TL at *p* < .05 with a FWE whole‐brain corrected peak‐level and cluster size (*k*) > 20, applying task‐specific masks. In our previous article (Powell et al., 2018), we found a positive effect of lithium medication on TL, for that reason we include lithium status in all subsequent analyses. Additionally, a recent meta‐analysis study by Rao et al. (2016) showed that TL is decreased in medicated patients with antipsychotics. Thus, age and BPRS scores were included as covariates in all models, alongside sex, subject group, lithium and antipsychotic status, and family relatedness included as fixed factors.

### 2.8 Dynamic causal modeling (effective connectivity)

For the facial affect recognition paradigm, previous studies implicate the inferior occipital gyrus (IOG), fusiform gyrus (FG), AMG, and ventral prefrontal cortex (VPFC), most consistently identified in the right hemisphere (Dima, Stephan, Roiser, Friston, & Frangou, 2011; Fairhall & Ishai, 2007; Torrisi, Lieberman, Bookheimer, & Altshuler, 2013). In our previous studies, the strategy for determining the most parsimonious model for facial affect processing employing the same participants was detailed (Dima et al., 2011, 2013; Dima, Roberts, & Frangou, 2016). In summary, we produced a basic 4‐node DCM in the right hemisphere with endogenous connections between volumes of interest (VOI) specified in the IOG, FG, AMG, and VPFC. The main effect of “all faces” was modeled as driving input to the IOG (Supporting Information Figure S1). We then created all possible models derived through permutation of condition‐specific responses (affective faces) on the forward coupling strength toward the VPFC.

Seven models were produced for the facial affect recognition paradigm (Supporting Information Material; Supporting Information Table S2 and Supporting Information Figure S1). To summarize the strength of effective connectivity and quantify its modulation, we used random effects Bayesian Model Averaging (BMA) to obtain average connectivity estimates across all models for each participant (Penny et al., 2010; Stephan et al., 2010). BMA DCM connections (n = 10) and modulations (n = 3) were extracted and tested separately using multivariate analysis of covariance models.

### 2.9 Effect of TL and PRS‐TL on effective connectivity

To test the effect of TL on effective connectivity (DCM connections and modulations) they were entered into a multivariate model with age, sex, group (BD patients, first‐degree healthy relatives, and unrelated healthy controls), BPRS scores, lithium status and relatedness as covariates. Since we are conducting 10 analyses for the connections and 3 for the modulations, we correct *p*‐values for multiple testing using false discovery rates (FDR) implemented in Matlab using the Benjamini and Hochberg (1995) procedure. The same analysis path was repeated for PRS‐TL.

## 3 RESULTS

### 3.1 Telomere length, behavioral task performance and medication

We used a univariate generalized linear model to compare patients, relatives, controls on TL, with age and sex as covariates. There was no significant effect of group on TL in the current sample (*F* = 0.94, *p* = .39; Table 1). No other significant correlations were found between TL and symptom severity (based on the total score of the HDRS, YMRS, and BPRS), age of onset, duration of illness, number of depressive, and manic episodes (*p* > .13).

There was a main effect of group on response time (*p* = .004), with patients being slower than the other two groups (*p* < .007; Table 1). Patients' medication type and dose did not affect their performance on the task (all *p* > .40). No significant correlations were found between TL and task performance (accuracy, reaction time).

We examined the effect of medication (on lithium vs. not on lithium, on antidepressants vs. not on antidepressants; typical antipsychotic, atypical antipsychotic, none; carbamazepine, lamotrigine, sodium valproate, none) on telomere length in patients with BD. We first tested the effect of each individual class and then considered all classes together. We found no effect of lithium (*t*_39_ = 1.19, *p* = .22), of antidepressants (*t*_39_ = 0.49, *p* = .68), antipsychotics (*F*_3, 38_ = 0.10, *p* = .89), or anticonvulsants (*F*_4, 37_ = 1.15, *p* = .34). When all medications and their interactions were considered, the main effects and interactions between medication classes were not significant (*p* > .53).

### 3.2 Facial‐affect brain activation

In all participants, activation in the affect > neutral faces contrast was found in the visual association and prefrontal cortical areas (Supporting Information Table S3). A group effect in the contrast affect > neutral faces was noted in the right ventral ACC and right superior frontal gyrus, where patients showed, respectively, increased and decreased activation compared with their relatives and unrelated controls (Supporting Information Table S4). For more details please see Dima, Roberts, & Frangou, 2016.

### 3.3 Effect of telomere length on face‐related activation

**Figure 1:**
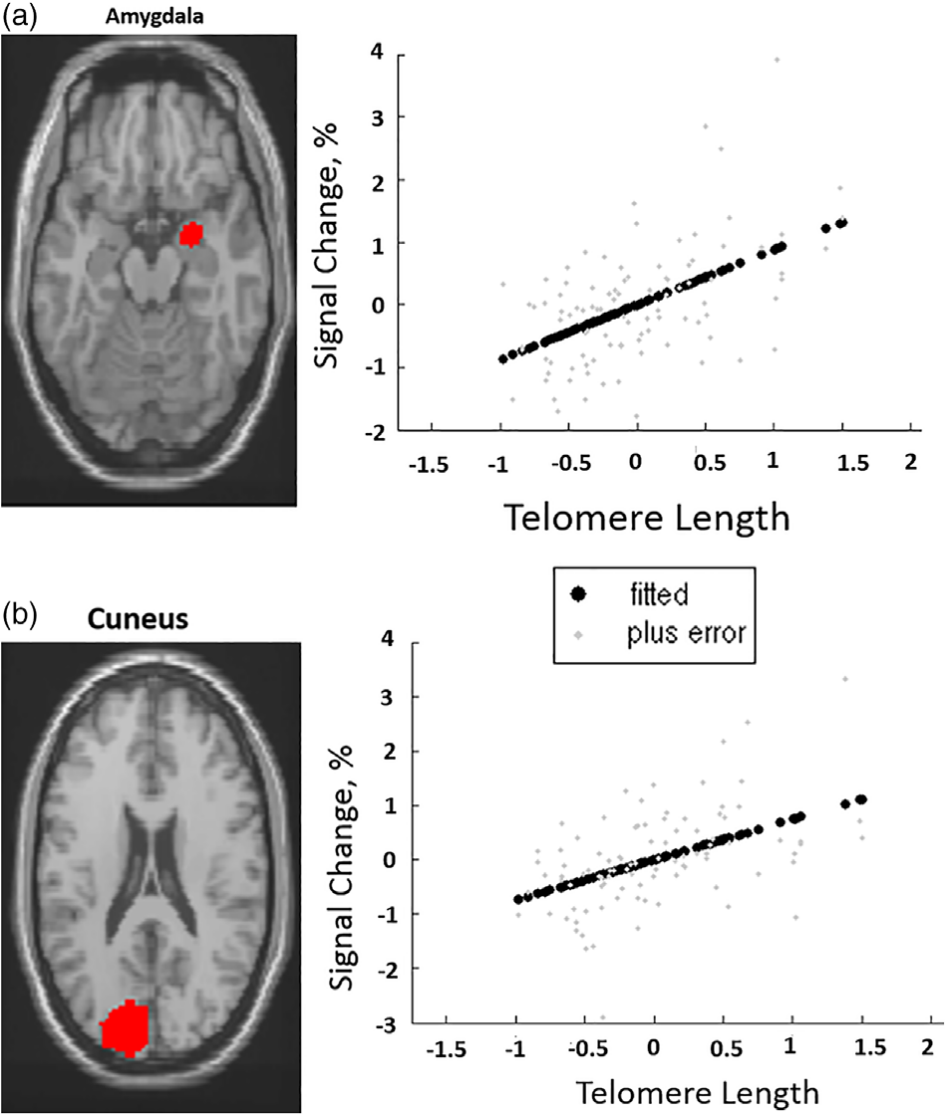
Telomere length positively correlated with task‐related activation in the (a) Amygdala, (b) Cuneus. “Fitted” response shows cross‐subject average and “plus error” shows individual subject's contrast values [Color figure can be viewed at https://wileyonlinelibrary.com]

Only significant positive correlations were found between TL and face‐related activation in the amygdala and cuneus (Figure 1; Table 2). There was no effect of group on these brain areas or an interaction between TL and group.

**Table 2:** Peak coordinates of significant positive correlations between telomere length and task related activation (n = 112; all *p* < .05 FWE, *k* > 20)

Region	Laterality	Brodmann area	MNI coordinates			Cluster size (*k*)	*z*‐value
			*x*	*y*	*z*		
*Facial affect recognition paradigm (affect > neutral faces)*							
Amygdala	Right	N/A	28	−2	−16	60	5.13
Cuneus	Left	18	−14	−86	26	28	4.62

FEW, family wise error; MNI, Montreal Neurological Institute.

Since, both age and TL are included in the regression models, and there is a potential for collinearity we performed additional analysis excluding age as a covariate from the regression model (the results were almost identical; Supporting Information Table S5) and tested the effect of age on face‐related brain activation (not significant, *p* > .05).

### 3.4 Effect of telomere length on effective connectivity

There was no effect of TL on effective connectivity (DCM connections; *F* = 0.98; *p* = .482). However, there was a main effect of TL on *facial affect* modulation on the three forward connections toward the VPFC (*F*_3,109_ = 2.25, *p* = .035). TL positively influenced the *facial affect* modulation from the IOG to the VPFC (*F*_1,112_ = 3.14; *p*_FDR_ = .048). Groups' differences in effective connectivity are described in Supporting Information Material.

### 3.5 Polygenic risk score telomere length (PRS‐TL)

High resolution polygenic risk scoring revealed that a *p*‐value threshold of *p* = .011 within our base dataset (TL GWAS), consisting of 1,793 SNPs, predicted 3.25% of the variance in TL within VIBES (*p* = .036). We subsequently utilized this as our instrumental variable, and outputted individualized PRS‐TL within VIBES at this threshold.

**Figure 2:**
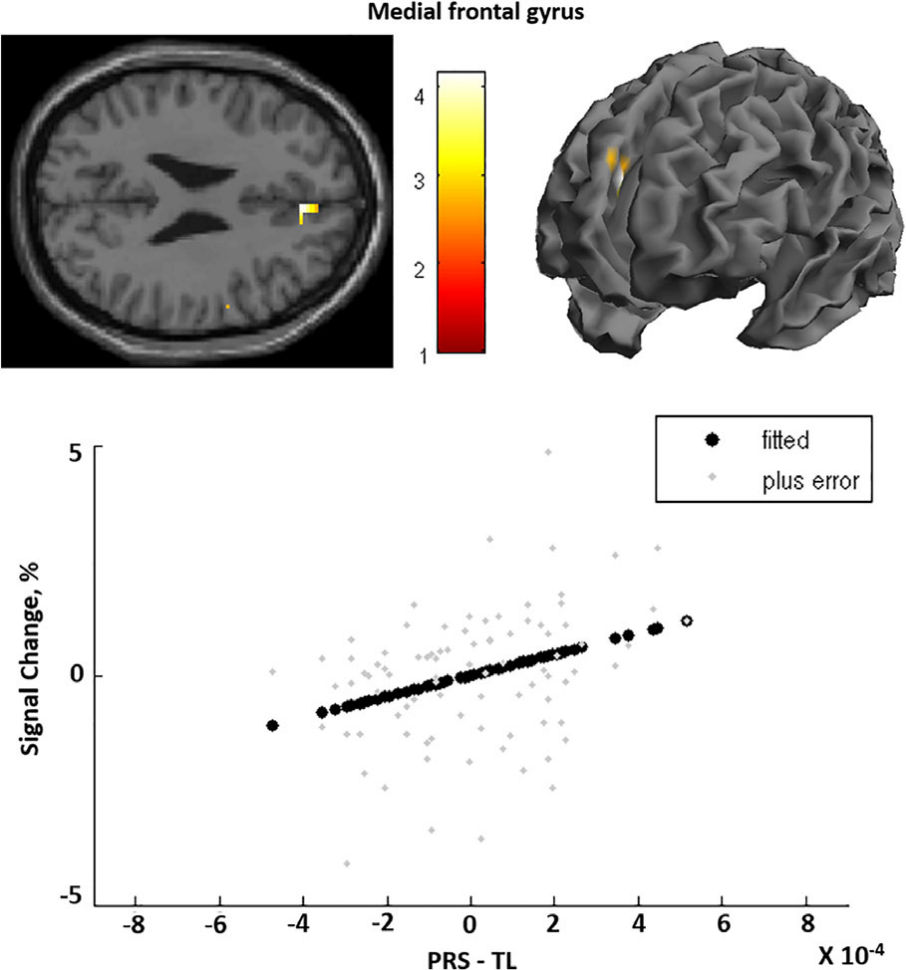
Association between polygenic risk score for telomere length (PRS‐TL) and activation in the right medial frontal gyrus. “Fitted” response shows cross‐subject average and “plus error” shows individual subject's contrast values [Color figure can be viewed at https://wileyonlinelibrary.com]

Testing PRS‐TL on facial affect brain activation, we find a positive correlation between PRS‐TL and the medial prefrontal gyrus (mPFC) (*x* = 4, *y* = 40, *z* = 24; *z*‐score = 4.84; *k* = 42 voxels; Figure 2). There was no interaction between PRS‐TL and TL on face‐related activation while performing the facial affect recognition task that survives FWE; even when we lower the threshold to *p* < .001 (uncorrected) there is no interaction. There was no effect of PRS‐TL on effective connectivity, intrinsic connections or facial affect modulation and no interaction of PRS‐TL with TL could be detected on connectivity (*p* > .31).

## 4 DISCUSSION

This is the first study to‐date to demonstrate a link between TL and emotional brain activity and connectivity, as well as a link between genetic risk for TL and emotional brain activation. First, TL was positively associated with activation in the amygdala and cuneus, and with connectivity between the posterior regions of the face network and the VPFC, during a task where participants were asked to label facial emotions. Second, the cumulative impact of risk‐alleles for TL, expressed as a polygenic risk score (PRS‐TL), showed a positive association with mPFC activation during facial affect.

### 4.1 The effect of TL on the facial affect‐processing network

Our results demonstrated that longer TLs are significantly associated with activity in the amygdala and cuneus when participants are asked to label facial emotions. The amygdala is central to emotion, involved in detecting the valence and intensity of expressed emotions (Whalen et al., 2009), especially negative emotion and its volume has also been positively associated with TL (King et al., 2014). It is thought to enhance the mental representation of other states such as those we perform here in labeling the emotion in strangers' faces (Ashworth et al., 2001). When considering TL as a state biomarker that captures the detrimental effects of age, cortisol, and oxidative stress, the result does complement previous findings, whereby longer TL marks healthier brain cells that retain the ability to generate action potentials (Uttara, Singh, Zamboni, & Mahajan, 2009). Furthermore, we know from rodent studies that the amygdala is a brain region particularly sensitive to the effects of oxidative stress, and consequently long buccal TL could represent an individual's reduced exposure to reactive oxygen species, and thus more functional cells in this brain region (Cano‐Europa et al., 2008).

In terms of a link with mood disorder pathology, longer TL being associated with increased amygdala activation is perhaps a little surprising, as mood disorders are generally associated with shorter telomeres and have previously been associated with amygdala hyper‐reactivity (Hamilton et al., 2012; Wegbreit et al., 2014). Consequently, it could be that mood disorders associated with amygdala hyper‐reactivity represent a subtype which is not usually marked by shorter TL, and so further work will be needed to test whether TL may be useful in differentiating patients with high and low amygdala activation in response to emotional stimuli. Alternatively, higher amygdala activity may represent a compensatory response, rather than pathological feature of mood disorders. In which case, higher amygdala activity (and longer TL) would be marking a healthy response to brain pathology. Future studies should investigate if amygdala hyperactivity in patients reflects amygdala dysfunction, by testing whether it is associated with behavioral deficits and poor clinical outcome, or whether it reflects a compensatory mechanism (Kim et al., 2012).

Perhaps more in keeping with the literature on mood disorders was the finding that longer TLs were positively associated with higher activation in the cuneus during emotion labeling. Our results add support to the notion of increased involvement of the visual cortices when attention is directed to the emotional valence of the stimuli (Dima et al., 2011; Gregoriou, Gotts, Zhou, & Desimone, 2009). Research until now has focused primarily on amygdala activation and its connectivity with bottom‐up and top‐down brain areas during the categorization of affective faces. However, recently equal attention has been drawn to the occipital lobe and its coupling to the VPFC as it seems that this coupling is a critical component of the brain network processing faces (Dima et al., 2011; Pessoa & Adolphs, 2010; Piech et al., 2010; Tsuchiya, Moradi, Felsen, Yamazaki, & Adolphs, 2009). The finding that increased cuneus activation is associated with longer TL is one which is complemented by our connectivity results that showed that the facial affect information forwarded from the IOG to the VPFC is significantly increased with longer TLs. Similarly, in brain tissue, no decrease in TL has been observed in studies quantifying telomere length in the occipital cortex (Teyssier, Ragot, Donzel, & Chauvet‐Gelinier, 2010) of subjects with psychiatric disorders and Holstege et al. (2014) showed that there is a differential effect of aging on brain tissues taken from the same individual, where the occipital cortex has the longest telomeres compared with all other brain areas. Our results contribute to this discussion by highlighting a potential role of TL in the occipital lobe, and by supplementing the current belief that TL in the occipital lobe is less susceptible to the effects of age and disease, while playing a role in emotional processing.

### 4.2 The effect of PRS‐TL on the facial affect‐processing network

When we perform the genetic analysis, we find a positive association between PRS‐TL and mPFC activation during facial affect labeling. The mPFC has been found to play a critical role in emotion facial discrimination (Winston, O'Doherty, & Dolan, 2003), emotional appraisal, expression and regulation (Etkin, Egner, & Kalisch, 2011) and memory for emotional facial expressions (Mattavelli, Cattaneo, & Papagno, 2011). Neuropsychological studies support this by demonstrating that patients with mPFC damage are impaired in recognizing emotional expressions and this deficit is associated with abnormal social behavior (Mah, Arnold, & Grafman, 2005) and reduced emotional responsiveness (Heberlein, Padon, Gillihan, Farah, & Fellows, 2008).

In animal work, studies working on TL in rats have found that female rats exposed to nurturing foster care outside of the homecage exhibited longer TL in the mPFC than females exposed to maltreatment or normal care (Asok, Bernard, Rosen, Dozier, & Roth, 2014). Similarly, prenatally‐stressed animals had shorter telomeres than controls in the mPFC (Blaze et al., 2017). Pivotally, a study using mice deficient for Tert, a key component of the enzyme telomerase responsible for the maintenance of telomere length, demonstrated the requirement of this telomere‐regulating enzyme for mood stability (Zhou, Wu, et al., 2016). Specifically, they showed that the re‐expression of Tert in the mPFC rescued the depressive phenotype of Tert^−/−^ mice, thus revealing a novel role of Tert in emotional control in the mPFC (Zhou, Ning, et al., 2016). Our results provide empirical support for the role of the PRS‐TL in the mPFC and further suggest that TL could be a mediator of emotional processing in the mPFC. Further studies will now need to understand why the mPFC is selectively affected by PRS‐TL, as opposed to other brain regions.

### 4.3 Considerations and conclusions

There are three main limitations of the current study which should be acknowledged. First, DNA used to measure TL was ascertained from buccal swabs rather than from brain tissue, meaning it may have useful predictive biomarker properties, but may lack construct validity (Powell, Fernandes, & Schalkwyk, 2012). Subsequently, it remains unclear to what extent peripheral TL correlates with brain‐region specific TL, and what precise neural mechanisms contribute to the association between TL and functional activity. However, previous studies have found that TL assessment using peripheral DNA is an acceptable surrogate since genetic influences on the regulation of TL appear tissue independent (Dlouha, Maluskova, Kralova Lesna, Lanska, & Hubacek, 2014; Friedrich et al., 2000). Furthermore, there is some animal work (Zhou, Wu, et al., 2016) which provides clues about connections between TL and the brain, but far more research is needed. Second, as TL changes over time, future studies incorporating longitudinal designs are needed to test whether the engagement of telomere‐eroding behaviors (e.g., smoking) or protective behaviors (e.g., exercise) impact on the functional processing of emotions. Third, TL likely represents a marker sensitive to the effects of a wide variety of environmental stress, and so further work will be needed to understand what factors affecting TL are particularly important in modulating brain activity (e.g., cortisol levels, diet, levels of reactive oxygen species, inflammatory cytokine levels) (Kordinas, Ioannidis, & Chatzipanagiotou, 2016; Lin, Epel, & Blackburn, 2012; Révész, Milaneschi, Verhoeven, & Penninx, 2014).

To the best of our knowledge, this study provides the first evidence linking TL and genetic risk for TL to brain activation and connectivity while categorizing emotional faces. We demonstrated that TL is associated with brain activity in regions known to be involved in emotional processing and are part of the facial‐affect processing network. Increased activation in amygdala and cuneus during emotional processing is predicted by longer TL. The current results also indicate the effect of genetic load for TL on brain function affects a key region for emotion, the mPFC. It is worth noting that shorter TL do not equate to developing a mood disorder, BD (Martinsson et al., 2013; Simon et al., 2006) or major depression disorder (Schaakxs, Verhoeven, Voshaar, Comijs, & Penninx, 2015; Vincent et al., 2017), rather TL may represent one of several pathways that increase vulnerability to mood disorders, through differential emotional processing. Future imaging telomere studies with larger and longitudinal samples would be uniquely informative in mapping the spatial distribution of TL on brain processes during emotional processing as patients and healthy individuals age, and may lead to the discovery of a biological mechanism linking TL maintenance with emotional brain function and the regulation of neural networks. TL could prove to be a faster and cheaper way of subtyping mood disorders based on their predicted emotional responses.

## Supporting information

Table S1 Sample Characteristics (N = 217)
Table S2. Model specification for the face affect paradigm
Table S3. Peak coordinates of task related activations in the entire sample (n = 112; all *p* < .05 FWE, k > 20)
Table S4. Brain regions showing significant effects of group in the facial affect recognition task (all p < .05 FWE, k > 20)
Table S5. Peak coordinates of significant positive correlations between telomere length and task related activation (n = 112; all p < .05 FWE, k > 20)
**Supplemental Figure 1. Seven dynamic causal models for the face affect paradigm for bipolar disorder patients (BD), their resilient relatives and healthy individuals.** The model is comprised of four brain areas specified with bidirectional endogenous connections between all regions (inferior occipital gyrus = IOG, fusiform gyrus = FG, amygdala = AMG, ventral prefrontal cortex = VPFC; all located in the right hemisphere) and with a driving input of “all faces” into the IOG. Green lines represent the affect faces modulation.
