# Supplementary material for: Telomere length as a predictor of emotional processing in the brain
Source: Hum Brain Mapp. 2018 Dec 4;40(6):1750–9. doi: 10.1002/hbm.24487 (PMC6492163; doi:10.1002/hbm.24487)
Supplement: Supplementary file 1 — Table S1 Sample Characteristics (N = 217) Table S2. Model specification for the face affect paradigm Table S3. Peak coordinates of task related activations in the entire sample (n = 112; all p < .05 FWE, k > 20) Table S4. Brain regions showing significant effects of group in the facial affect recognition task (all p < .05 FWE, k > 20) Table S5. Peak coordinates of significant positive correlations between telomere length and task related activation (n = 112; all p < .05 FWE, k > 20) Supplemental Figure 1. Seven dynamic causal models for the face affect paradigm for bipolar disorder patients (BD), their resilient relatives and healthy individuals. The model is comprised of four brain areas specified with bidirectional endogenous connections between all regions (inferior occipital gyrus = IOG, fusiform gyrus = FG, amygdala = AMG, ventral prefrontal cortex = VPFC; all located in the right hemisphere) and with a driving input of “all faces” into the IOG. Green lines represent the affect faces modulation. [file HBM-40-1750-s001.docx]

**Supplemental Material**

**Methods**

*Dynamic Causal Modelling*

DCM tests a set of models and, through Bayesian model selection, provides evidence in favor of one model, relative to others. In DCM regional blood-oxygen-dependent responses are modeled by a bilinear differential equation that describes how the underlying neural states change as a function of endogenous connections between regions (referred to as nodes), modulatory effects on these connections, and driving inputs (Friston et al., 2003). Endogenous connections refer to the coupling strength between nodes in the absence of any input to the network (task-independent). Modulatory effects refer to task-dependent changes in coupling strength. The driving input models how task-related information enters the network. For each task, we defined the relevant model space (i.e., the set of models that are plausible) based on current best evidence regarding the neural circuitry that supports facial affect recognition and working memory.

For the *facial affect recognition* paradigm, the spatial part of the basic 4- node model was specified as four VOIs, each of 5mm radius, centered on the coordinates of the group maxima of the contrast affective > neutral faces from the entire study sample: IOG (x=44, y=-76 , z=-1), FG (x=24, y=-55, z=-7), AMG (x=16, y=-3, z=-13) and VPFC (x=51, y=25, z=-6).

The spatial models were further refined for each participant based on the participant-specific maxima that were: (i) within 4 mm from the group maxima, (ii) within the same anatomical regions, as defined by the PickAtlas toolbox (http://www.nitrc.org/projects/wfu_pickatlas/) and (iii) adjusted using the effect of interest F-contrast. Regional time series were summarized with the first eigenvariate of all activated (at p<0.01) voxels within participant-specific VOIs.

Friston KJ, Harrison L, Penny W. Dynamic causal modelling. *Neuroimage* 2003; **19**: 1273-1302.

**Results**

*Group effects of effective connectivity*

Across all seven models, there was a significant effect of group on the reciprocal endogenous connectivity between the IOG and the FG, which was higher in healthy relatives compared with both other groups (P < 0.04). Effective connectivity in BD patients compared with healthy individuals was associated with reduced connectivity between IOG and VPFC (P = 0.02) but increased between AMG and VPFC (P = 0.03) (for more details please see Dima et al., 2016).

Dima D, Roberts RE, Frangou S. 2016. Connectomic markers of disease expression, genetic risk and resilience in bipolar disorder. Transl Psychiatry. 6: e706.

*Effect of telomere length on face-related activation*

Additional analysis for performed to test the effect of collinearity between age and TL in the regression models. We performed a second-level analysis of regression in SPM8 to identify clusters of correlation with age at P < 0.05 with a FWE whole-brain corrected peak-level and cluster size (k) > 20, applying task-specific masks. BPRS scores were included as covariates in all models, alongside sex, subject group and family relatedness included as fixed factors. No significant clusters were identified, either positive or negative.

We also performed a second-level analysis of regression in SPM8 to identify clusters of correlation with TL without including age as a covariate. BPRS scores were included as covariates in all models, alongside sex, subject group, lithium and antipsychotic status and family relatedness included as fixed factors.

The results were almost identical to the ones included in the main manuscript, please see below (Table S5).

| **Table S1: Sample Characteristics (N=217)** | | | |
| --- | --- | --- | --- |
|  | **Patients**  **N=63** | **Relatives**  **N=74** | **Unrelated**  **Participants**  **N=80** |
| **Age (years)^a^** | 44.04 (10.33) | 34.50 (12.85) | 39.71 (14.82) |
| **Sex, n (% male)** | 30 (47.61) | 33 (44.59) | 36 (45) |
| **IQ** | 118.80 (18.24) | 115.31 (16.71) | 121.82 (19.88) |
| **Hamilton Depression Rating Scale^b^** | 3.88 (4.67) | 0.66 (1.53) | 0.17 (0.61) |
| **Young Mania Rating Scale^b^** | 1.17 (2.29) | 0.14 (0.72) | 0.15 (0.45) |
| **Age of onset of Bipolar Disorder (years)** | 25.50 (8.38) | n/a | n/a |
| **Any Medication, n (%)^c^** | 59 (93.65) | 15 (20.27) | n/a |
| **Lithium (n)** | 28 (44.44) | 0 | n/a |
| **Any Antidepressant (n)^d^** | 31 (49.20) | 15 (20.27) | n/a |
| **Any Antipsychotic (n)^e^** | 24 (38.09) | 0 | n/a |
| **Any Anticonvulsant (n)^f^** | 26 (41.26) | 0 | n/a |
| All continuous variables are shown as mean (standard deviation); Intelligence Quotient (IQ) was derived from the Wechsler Adult Intelligence Scale-Revised; WMS= Wechsler Memory Scale-III; VPA=Verbal Paired Associates; Scaled scores reported for both WMS-VPA measures  ^a^ Relatives <Patients and Controls; p<0.03; ^b^ Patients> Relatives, Controls, all p<0.0001; ^c^ 59 patients were prescribed more than one psychotropic; ^d^ all antidepressants prescribed were serotonin reuptake inhibitors; ^e^ all but 3 antipsychotics prescribed were second generation agents; ^f^ sodium valproate=14; carbamazepine=5; lamotrigine=2; combinations=5 | | | |

| **Table S2. Model specification for the face affect paradigm** | |
| --- | --- |
| **Model** | **Facial affect modulation on:** |
|  | |
| Model 1 | IOG → VPFC |
| Model 2 | FG → VPFC |
| Model 3 | AMG → VPFC |
| Model 4 | IOG → VPFC; FG → VPFC; AMG → VPFC |
| Model 5 | IOG → VPFC; FG → VPFC |
| Model 6 | FG → VPFC; AMG → VPFC |
| Model 7 | IOG → VPFC; AMG → VPFC |

**Table S3. Peak coordinates of task related activations in the entire sample (n = 112; all P < 0.05 FWE, k > 20)**

| **Region** | **Laterality** | **Brodmann Area** | **MNI Coordinates** | | | **Cluster size (k)** | **z-value** |
| --- | --- | --- | --- | --- | --- | --- | --- |
|  |  |  | **x** | **y** | **z** |  |  |
| **Facial affect recognition paradigm (affect > neutral faces)** | | | | | | | |
| Lingual Gyrus | Right | 18 | 10 | -76 | -2 | 635 | 7.7 |
| Middle Frontal Gyrus | Right | 9 | 34 | 14 | 32 | 555 | 6.96 |
| Inferior Frontal Gyrus | Right | 47 | 54 | 18 | -4 | 202 | 6.51 |
| Amygdala | Left | N/A | -24 | -2 | -16 | 57 | 6.21 |
| Middle Occipital Gyrus | Left | 18 | -32 | -88 | 2 | 34 | 5.99 |
| Inferior Occipital Gyrus | Left | 18 | -48 | -72 | -4 | 30 | 5.85 |
| Inferior Parietal Lobule | Left | 40 | -50 | -42 | 52 | 147 | 5.83 |
| Superior Parietal Lobule | Right | 7 | 36 | -62 | 54 | 72 | 5.82 |
| Superior Temporal Gyrus | Left | 38 | -52 | 12 | -10 | 84 | 5.74 |
| Inferior Temporal Gyrus | Right | 19 | 52 | -66 | -6 | 37 | 5.29 |
| FWE =family wise error; Montreal Neurological Institute=MNI | | | | | | | |

**Table S4. Brain regions showing significant effects of group in the facial affect recognition task (all p < 0.05 FWE, k > 20)**

| **Region** | **Laterality** | **Brodmann Area** | **MNI Coordinates** | | | **Cluster size (k)** | **z-value** |
| --- | --- | --- | --- | --- | --- | --- | --- |
|  |  |  | **x** | **y** | **z** |  |  |
| **Facial affect-recognition paradigm (affective > neutral faces)** | | | | | | | |
| Anterior Cingulate Gyrus | Right | 24 | 4 | 34 | 1 | 35 | 3.23 |
| Superior Frontal Gyrus | Right | 6 | 38 | 18 | 64 | 41 | 3.12 |
| FWE =family wise error; Montreal Neurological Institute=MNI | | | | | | | |

**Table S5. Peak coordinates of significant positive correlations between telomere length and task related activation (n = 112; all p < 0.05 FWE, k > 20)**

| **Region** | **Laterality** | **Brodmann Area** | **MNI Coordinates** | | | **Cluster size (k)** | **z-value** |
| --- | --- | --- | --- | --- | --- | --- | --- |
|  |  |  | **x** | **y** | **z** |  |  |
| **Facial affect recognition paradigm (affect > neutral faces)** | | | | | | | |
| Amygdala | Right | N/A | 28 | -4 | -18 | 52 | 5.27 |
| Cuneus | Left | 18 | -14 | -86 | 26 | 36 | 4.81 |
| FWE = family wise error; Montreal Neurological Institute = MNI | | | | | | | |

**Supplemental Figure 1.** **Seven dynamic causal models for the face affect paradigm for bipolar disorder patients (BD), their resilient relatives and healthy individuals.** The model is comprised of four brain areas specified with bidirectional endogenous connections between all regions (inferior occipital gyrus = IOG, fusiform gyrus = FG, amygdala = AMG, ventral prefrontal cortex = VPFC; all located in the right hemisphere) and with a driving input of ‘all faces’ into the IOG. Green lines represent the affect faces modulation.

**
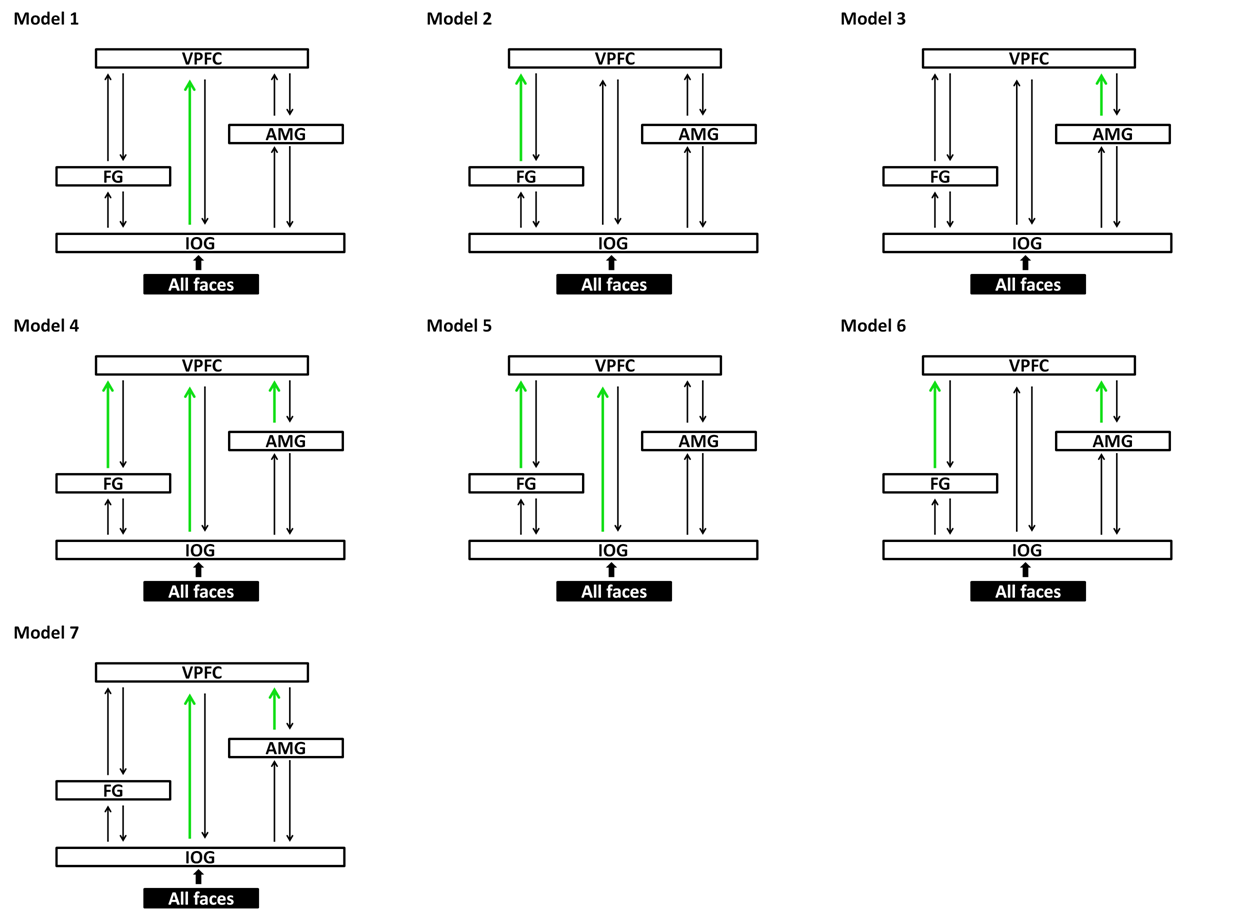
**
